# Anticancer and Differentiation Properties of the Nitric Oxide Derivative of Lopinavir in Human Glioblastoma Cells

**DOI:** 10.3390/molecules23102463

**Published:** 2018-09-26

**Authors:** Maria Sofia Basile, Emanuela Mazzon, Tamara Krajnovic, Dijana Draca, Eugenio Cavalli, Yousef Al-Abed, Placido Bramanti, Ferdinando Nicoletti, Sanja Mijatovic, Danijela Maksimovic-Ivanic

**Affiliations:** 1 Department of Immunology, Institute for Biological Research “Sinisa Stankovic”, Belgrade University, Bulevar despota Stefana 142, 11060 Belgrade, Serbia; sofiabasile@hotmail.it (M.S.B.); tamara_krajnovic@yahoo.com (T.K.); dracadiana@gmail.com (D.D.); sanjamama@ibiss.bg.ac.rs (S.M.); nelamax@ibiss.bg.ac.rs (D.M.-I.); 2 Department of Biomedical and Biotechnological Sciences, University of Catania, Via S. Sofia 89, 95123 Catania, Italy; 3 IRCCS Centro Neurolesi Bonino Pulejo, Strada Statale 113, C.da Casazza, 98124 Messina, Italy; emanuela.mazzon@irccsme.it (E.M.); eugeniocavalli9@hotmail.it (E.C.); placido.bramanti@irccsme.it (P.B.); 4 Center for Molecular Innovation, The Feinstein Institute for Medical Research, 350 Community drive, Manhasset, NY 11030, USA; yalabed@northwell.edu

**Keywords:** glioblastoma, HIV protease inhibitors, Lopinavir, Lopinavir-NO, nitric oxide

## Abstract

Glioblastoma (GBM) is the most frequent and deadly form of primary malignant brain tumor among adults. A promising emerging approach for GBM treatment may be offered from HIV protease inhibitors (HIV-PIs). In fact, in addition to their primary pharmacological activity in the treatment of HIV infection, they possess important anti-neoplastic effects. According to previous studies, the addition of a nitric oxide (NO) donating group to parental compounds can reduce their toxicity and enhance the anticancer action of various compounds, including HIV-PIs. In this study we compared the effects of the HIV-PI Lopinavir (Lopi) and of its NO-derivative Lopinavir-NO (Lopi-NO) on the in vitro growth of LN-229 and U-251 human GBM cell lines. Lopi-NO reduced the viability of LN-229 and U-251 cells at significantly lower concentrations than the parental drug. In particular, Lopi-NO inhibited tumor cell proliferation and induced the differentiation of U-251 cells toward an astrocyte-like phenotype without triggering significant cell death in both cell types. The anticancer effect of Lopi-NO was persistent even upon drug removal. Furthermore, Lopi-NO induced strong autophagy that did not appear to be related to its chemotherapeutic action. Overall, our results suggest that Lopi-NO could be a potential effective anticancer drug for GBM treatment.

## 1. Introduction

Glioblastoma (GBM) is the most frequent and deadly form of primary malignant brain tumor among adults [1]. According to the World Health Organization (WHO), GBM is classified as a grade IV astrocytic tumor [2]. GBM represents a major unmet medical need, showing an average age-adjusted incidence rate of 0.59 to 3.69/100,000 cases per year and a median survival of 14.6 months after diagnosis [3]. The standard of care treatment for GBM is surgery, coupled with radiotherapy and chemotherapy with Temozolomide [4]. Several novel therapeutic approaches are being studied that include nanotechnology (colloidal nanocarriers, liposomes, polymeric nanoparticles and lipid nanocapsules), immunotherapeutic approaches (vaccines, cell-based therapies, and immune checkpoint modulators) and strategies for effective brain drug delivery (tight junction opening, inhibition of efflux transporters present at the blood–brain barrier, use of chemically modified drugs, and craniotomy-based drug delivery) [5]. In addition, recent studies have highlighted a key role for abnormal function of both innate and adaptive immune system in the pathogenesis of GBM that include dysregulated production of anti-inflammatory cytokines such as interleukin (IL)-10 [6], IL-13 [7], and transforming growth factor (TGF)-beta [8], that are thought to be responsible for contribution to immune evasion of the tumor by polarizing macrophages from M1 to M2 phenotype and function [9]. However, the ability of polarizing macrophages toward a M2 phenotype is not only restricted to anti-inflammatory cytokines, dysregulated expression of the proinflammatory cytokines as macrophage migration inhibitory factor (MIF) and its homologue D-Dopachrome Tautomerase (DDT) and their receptors have also been observed in GBM [10] and it has been proposed that MIF may represent a useful diagnostic marker and therapeutic target for the use of anti-MIF tailored inhibitors for some cases of GBM [5]. In turn M1 to M2 polarization may enhance the expression and functional activity of events and may create a milieu favoring immune escape also from upregulated expression of immune checkpoint inhibitors such as cytotoxic T lymphocyte-associated antigen 4 (CTLA4) and programmed cell death (PD)-1 that ultimately generate a local pro-oncogenic milieu favoring immune evasion and growth of GBM [9].

These concepts have translated well into the clinical setting with studies aimed at restoring the defective immune function by interfering with PD1/PD-ligand(PDL)-1 signaling pathway and by chimeric antigen receptor T (CAR-T) cell therapy [11,12].

In addition to these treatments, another class of drugs that is attracting increasing interest for their anticancer properties and could also be used for GBM is represented from HIV protease inhibitors (HIV-PIs). HIV-PIs, along with reverse transcriptase inhibitors and/or an integrase inhibitor, are included in highly active antiretroviral therapy, the ongoing most efficient AIDS treatment [13]. These potent antiretroviral drugs block HIV replication through selective binding to the catalytic site of the HIV protease [14]. Currently, the US Food and Drug Administration (FDA) has approved ten HIV-PIs: Saquinavir (Saq), Ritonavir, Indinavir, Nelfinavir, Amprenavir, Lopinavir (Lopi), Atazanavir, Fosamprenavir, Tipranavir, and Darunavir [13].

However, converging and increasing preclinical and clinical evidence indicates that beside their antiretroviral action, HIV-PIs exert also an anti-neoplastic activity that make them capable of inhibiting the growth of tumor and cancer stem cells (CSCs) as well as to suppress angiogenesis [15]. That HIV-PIs could have anticancer actions was first hypothesized observing the significant lower rate of HIV associated cancers (Kaposi’s sarcoma and some non-Hodgkin lymphomas) in HIV-infected patients treated with these drugs [16]. 

In particular, several preclinical and clinical studies have investigated the potential anticancer effects and the possible mechanisms of action of HIV-PIs in GBM. The most studied HIV-PIs in this setting are Nelfinavir and Ritonavir. Nelfinavir has been shown to reduce Akt phosphorylation and improve radio- and chemosensitization in GBM cells [17], to cause malignant glioma death through the induction of endoplasmic reticulum stress response both in vitro and in vivo [18] and to inhibit vascular endothelial growth factor (VEGF)/hypoxia-inducible factor (HIF)-1alpha expression and angiogenesis in GBM cells [19]. Furthermore, Nelfinavir, along with Captopril and Disulfiram, could be effective in GBM treatment by inhibiting matrix metalloproteinases [20]. A phase I trial [21] showed that the combination of Nelfinavir with Temozolomide and radiotherapy was well tolerated in patients with GBM. On the other hand, Ritonavir exhibits a cytostatic and cytotoxic action on glioma cells through the inhibition of the chymotrypsin-like activity of the proteasome [22] and exerts synergistic activity with Aprepitant in inhibiting glioma cell growth by decreasing Akt signaling [23]. Moreover, Ritonavir and Disulfiram inhibit the proinflammatory cytokine IL-18 [24], which is involved in GBM cell migration [25]. A phase II trial [26] has shown that Ritonavir/Lopi were well tolerated in patients with progressive or recurrent high-grade gliomas (WHO grades 3–4). Lopi has also been described to exert an anti-neoplastic activity. In fact, Lopi could target CSCs [27], inhibit meningioma cell proliferation through Akt-independent pathway [28] and induce apoptosis of primary effusion lymphoma cells through blocking the nuclear factor (NF)-kB pathway [29].

However, although HIV-PIs may represent an effective alternative and/or additional anticancer strategy, these drugs have several dose-dependent side effects and relatively weak anticancer potency [15]. Hence, efforts to increase their anticancer potency with simultaneous reduction of doses are warranted. According to previous studies, the addition of a nitric oxide (NO) donating group could reduce toxicity and improve the anticancer action of various parental compounds, including nonsteroidal anti-inflammatory drugs (NSAIDS) [30] and HIV-PIs [15]. During these last years, our group has synthetized a NO-hybridized form of Saq, named Saq-NO, that has been shown to maintain the antiretroviral effect of Saq [31], and acquire antitumoral and immunomodulatory properties, along with reduced toxicity in vitro and in vivo [14,32,33,34,35]. 

Along this line of research we have also generated the NO-derivative of Lopi, named Lopi-NO, and have shown that it induced caspase-dependent apoptosis and markedly inhibited cellular proliferation on blood cancer cells with a superior anticancer action than its parental compound Lopi [36]. A previous study also showed that Lopi-NO exhibited significantly stronger immunomodulatory effects than Lopi both in vitro and in vivo [37].

These data prompted us to study in a head to head comparison the effects and the insights into anticancer mode of action of Lopi-NO vs. its parental compound Lopi on the in vitro growth of LN-229 and U-251 GBM cell lines.

The results of this study confirm that modified drug is more effective in reducing human GBM cell growth and acquired peculiar and more potent anticancer effects than Lopi.

## 2. Results

### 2.1. Lopi-NO Has Stronger Anticancer Action Than Lopi

**Figure 1:**
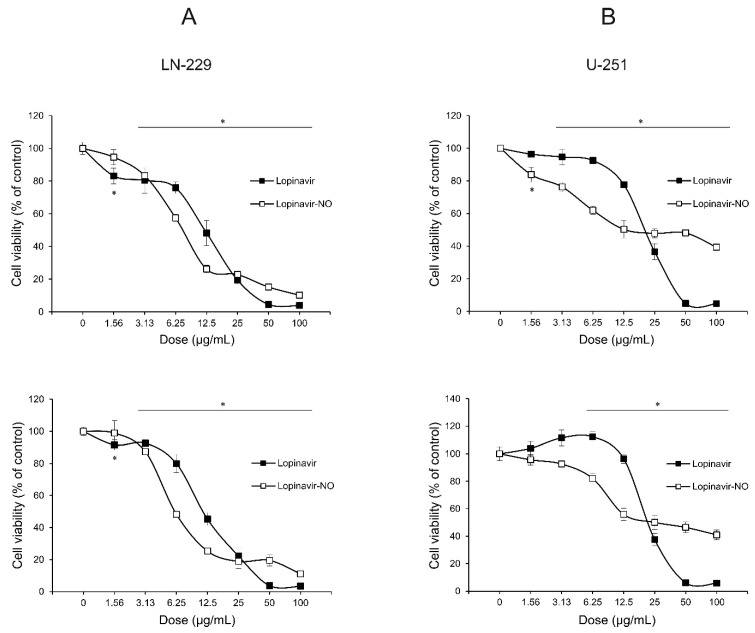
Lopinavir (Lopi) and Lopi-NO reduced the viability of glioblastoma (GBM) cell lines in a dose-dependent manner. LN-229 (**A**) and U-251 (**B**) cells were treated with a range of doses of Lopi and Lopi-NO for 48 h. Cell viability was determined by 3-(4,5-dimethylthiazol-2-yl)-2,5-diphenyltetrazolium bromide (MTT) (upper panel) and crystal violet (CV) assays (lower panel). The data are presented as percentage of control ± standard deviation (SD) from one representative out of three independent experiments. * *p* < 0.05 refers to untreated cultures.

To evaluate the sensitivity of the human GBM cell lines U-251 and LN-229 to Lopi and Lopi-NO, the cells were exposed to a range of concentrations of both compounds and after 48 h cell viability was measured by (3-(4,5-dimethylthiazol-2-yl)-2,5-diphenyltetrazolium bromide (MTT) and crystal violet (CV) assays. The results of both assays were well synchronized. As evidenced in Figure 1, both drugs expressed remarkable antitumor potential. However, while Lopi decreased the number of viable cells in concentration-dependent manner, Lopi-NO reached a plateau-effect. Calculated IC_50_ values revealed that despite initial difference in LN-229 vs. U-251 sensitivity to both compounds, Lopi-NO was more potent than Lopi (Table 1).

### 2.2. Lopi-NO Inhibited Tumor Cell Proliferation and Induced Strong Autophagy

**Figure 2:**
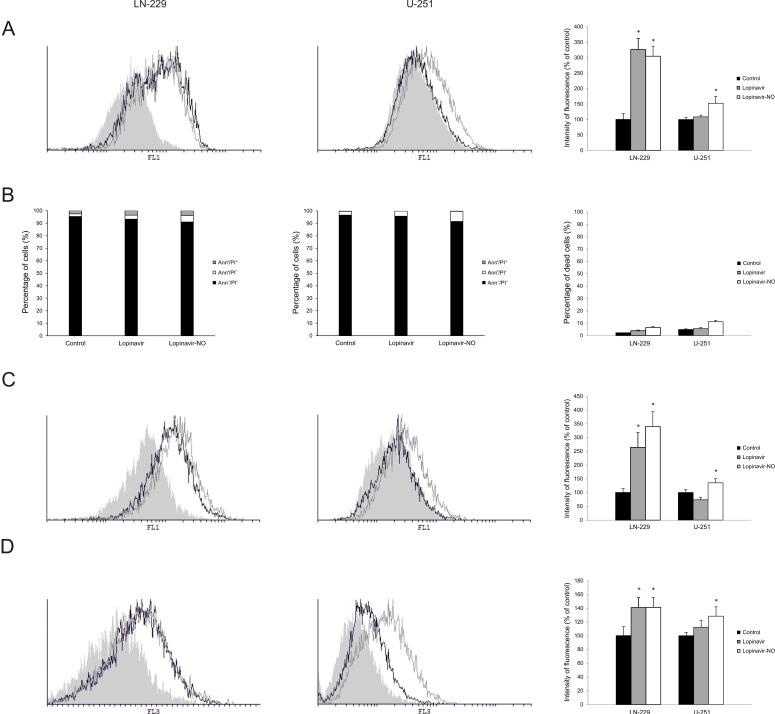
Lopi-NO induced inhibition of proliferation, insignificant apoptosis, and strong autophagy in GBM cell lines. GBM cells were treated with IC_50_ doses of both compounds and then subjected to (**A**) carboxyfluorescein diacetate succinimidyl ester (CFSE), (**B**) annexin V-FITC (AnnV)/Propidium Iodide (PI), (**C**) apostat, and (**D**) acridine orange staining. Cells were analyzed by flow cytometry (**A**–**D**) and representative histograms (left and middle panel) and charts of three independent experiments (right panel) are shown.

The observed plateau-effect in curves of viability of both cell lines suggested that Lopi-NO affected cell viability through downregulation of cell division rather than cell death. To confirm this, flow cytometric analyses of LN-229 and U-251 cells were performed after 48 h of the treatment with IC_50_ doses of either Lopi or Lopi-NO. In LN-229 cells, measurement of cellular proliferation, apoptosis, caspase activity, and presence of autophagy showed comparable mode of action of Lopi and Lopi-NO. In contrast, the same analyses performed on U-251 cells clearly differentiated the mode of action of Lopi and Lopi-NO. As presented on Figure 2, in both cell lines Lopi-NO affected cell division with minimal contribution of apoptotic cell death. Its application promoted caspase activation independently from moderate presence on Annexin V-FITC (AnnV)+/Propidium Iodide (PI)^−^ and AnnV+/PI^+^ cells. Accordingly, intensified presence of acidic vesicles as markers of autophagic process was detected. While Lopi-NO triggered strong autophagy in both cell lines, the parental compound Lopi only induced it in LN-229 but not in U-251. Taken together, these data indicate that NO hybridization improved anticancer properties of Lopi on both cell lines. For this reason, Lopi-NO was selected for further investigation.

### 2.3. Lopi-NO Triggered Permanent Change in GBM Cell Phenotype 

**Figure 3:**
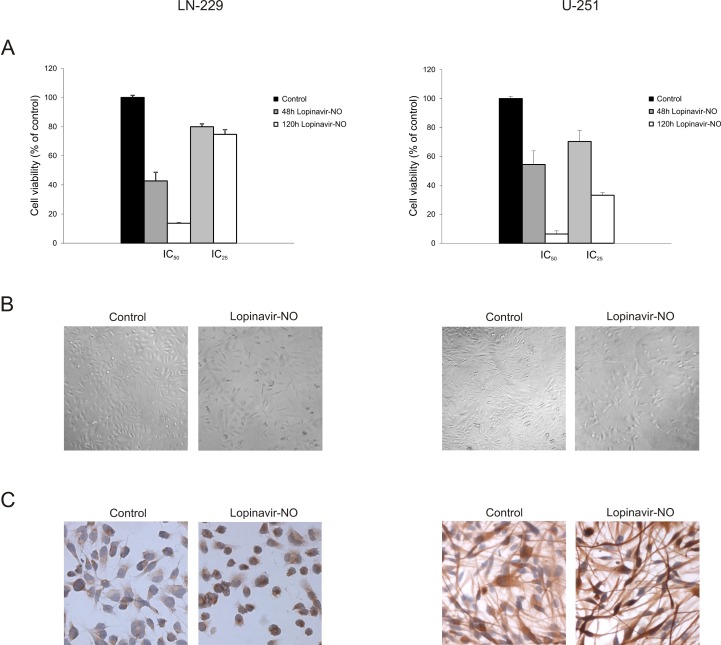
Lopi-NO induced persistent inhibition of proliferation accompanied with phenotype change. GBM cells were treated with IC_50_ and IC_25_ doses of tested compounds for 48 h, when the drug was removed and (**A**) cellular viability was estimated by CV test after additional 72 h of incubation. (**B**) Cell morphology was assessed by light microscopy after 48 h of incubation in the presence of IC_50_ of Lopi-NO (magnification 50×). (**C**) Glial fibrillary acidic protein (GFAP) expression in GBM cells after 48 h of incubation in the presence of IC_50_ of Lopi-NO (magnification 250×). * *p* < 0.05 in comparison to control.

To investigate whether the suppressed proliferation induced by Lopi-NO required the continuous presence of the compound, the cells were treated for 48 h with Lopi-NO, and after this period of time, the drug was removed and cell viability was measured with CV assay after additional 72 h of incubation. The results showed that a 48-h pulse with Lopi-NO was sufficient to permanently inhibit cell proliferation in both cell lines (Figure 3A). Persistent inhibition of proliferation was accompanied with decreased tumor cell number and change in cell shape and size in response to Lopi-NO treatment. This effect was more obvious in U-251 culture where almost all cells obtained spindle like phenotype with long tapering extensions (Figure 3B). This morphological transformation indicated that cells entered differentiation process. To prove this, glial fibrillary acidic protein (GFAP) expression, as a marker of maturation toward astrocytes, was evaluated by immunocytochemistry. In agreement with the literature [38], LN-229 initially did not express this protein either constitutively or after exposure to Lopi-NO. On the other hand, intensified expression upon the same treatment was determined in U-251 cells, confirming the astrocytic lineage differentiation (Figure 3C).

### 2.4. Autophagy Was Irrelevant for U-251 Differentiation

**Figure 4:**
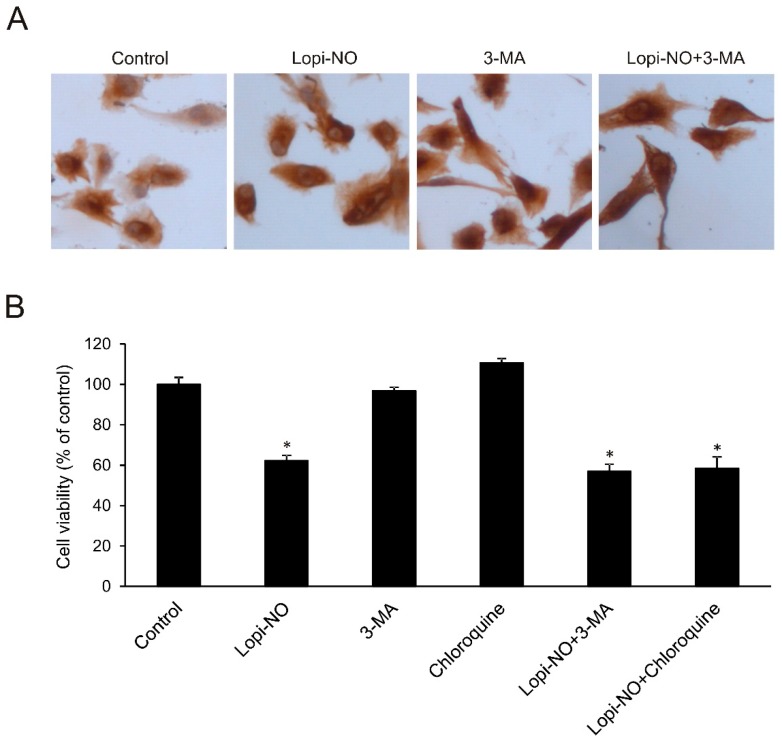
Autophagy is not relevant for differentiation of U-251 induced by Lopi-NO. Cells were treated with the IC_50_ value of Lopi-NO in the presence of autophagy inhibitor 3-methyladenine (3-MA) (1 mM) or chloroquine (20 μM) for 48 h and (**A**) GFAP expression by immunocytochemistry (magnification 320×) and (**B**) cellular viability by MTT test were estimated. * *p* < 0.05 refers to untreated cultures.

Since autophagy might be included in glioma cell differentiation, the possible involvement of this process in Lopi-NO triggered maturation of U-251 cells was evaluated in the presence of specific inhibitor, 3-methyladenine (3-MA). The results showed that inhibition of autophagy did not influence GFAP expression in cells treated with Lopi-NO (Figure 4A), confirming that autophagy did not contribute to differentiation of U-251 cells. To further define the role of autophagy, the cells were exposed to Lopi-NO alone or in combination with two different autophagic inhibitors such as chloroquine and 3-MA. Inhibition of autophagy by chloroquine is based on the elevation of the lysosomal pH, further fusion of autophagosome with lysosome, and subsequent proteolytic degradation while 3-MA suppresses the formation of autophagosomes by inhibition of phosphoinositide 3-kinase (PI3K)/Akt signaling pathway. The data showed that the viability of U-251 cells was not restored upon neutralization of autophagy (Figure 4B). On the other hand, in LN-229 the cotreatment with both autophagy inhibitors dramatically potentiated the anticancer action of Lopi-NO (Figure S1). In summary, autophagy seems to represent a counterregulatory response of the cells to the action of the drug.

### 2.5. Lopi-NO Promoted Oxidative/Nitrosative Stress

**Figure 5:**
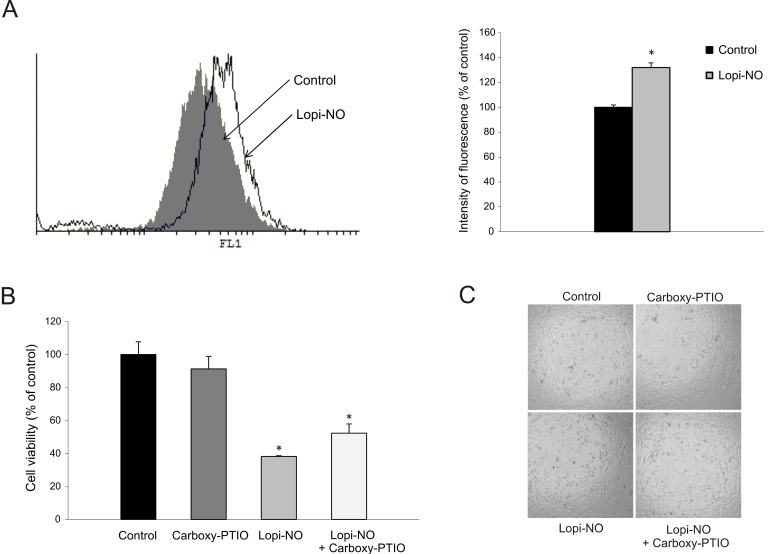
Lopi-NO induced reactive oxygen species (ROS)/reactive nitrogen species (RNS) production in U-251 cells. (**A**) Before treatment with IC_50_ dose of Lopi-NO for 48 h, cells were subjected to dihydrorhodamin 123 (DHR) staining and analyzed by flow cytometry. One representative histogram (left) and chart of three independent experiments (right) are shown. Cells were treated with Lopi-NO and/or carboxy-PTIO (20 µM) for 48 h and subjected to (**B**) CV staining and (**C**) light microscopy (magnification 40×). Data are presented as mean ± SD of three independent experiments. * *p* < 0.05 compared to untreated cultures.

To evaluate the influence of Lopi-NO on the level of reactive oxygen species (ROS)/reactive nitrogen species (RNS), cumulative production of these molecules was quantified using dihydrorhodamin 123 (DHR) indicator. After 48 h of incubation, significant enhancement in fluorescence intensity corresponding to the amount of radicals produced was determined (Figure 5A). Our unpublished data indicate that Lopi-NO releases NO inside the tumor cells. To define the contribution of NO release to drug toxicity, as well as cell morphology, the cells were exposed to intracellular NO scavenger, carboxy-PTIO. Neutralization of NO resulted in recovered viability of U-251 cells suggesting that NO released from the drug was, at least partly, responsible for its antitumor effect (Figure 5B). On the other hand, elimination of NO did not reflect on cell morphology indicating that this molecule was not crucial for the differentiation-inducing potential of the compound (Figure 5C).

### 2.6. Lopi-NO Antagonized Cisplatin Activity in Cotreatment

**Figure 6:**
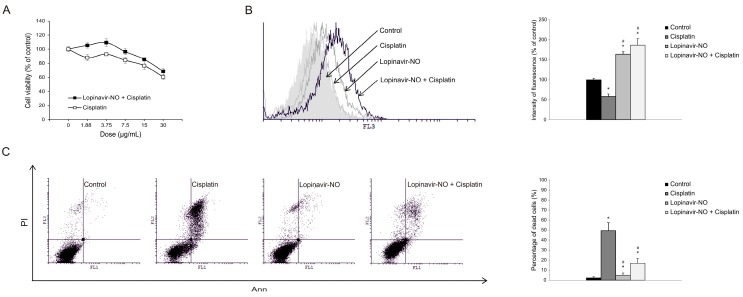
Lopi-NO antagonized Cisplatin toxicity in LN-229 cells. Cells were exposed to Cisplatin in the presence of IC_50_ value of Lopi-NO and (**A**) cellular viability by CV test, (**B**) autophagy by acridine orange staining, and (**C**) apoptosis by AnnV/PI staining were performed. * *p* < 0.05 in comparison tocontrol, ^#^ *p* < 0.05 in comparison to Cisplatin. Representative results of flow cytometry (B, C, left) and charts of three independent experiments (B, C, right) are shown.

Since a cytoprotective role of autophagy was defined upon Lopi-NO in both cell lines, it was interesting to evaluate eventual interference with standard chemotherapy. To this aim, the cells were exposed to Lopi-NO for 24 h and subsequently cotreated with Cisplatin. The data showed that cotreatment with Lopi-NO neutralized the effects of Cisplatin in LN-229 cells (Figure 6A). This was in concordance with the previously described strong cytoprotective effect of autophagy in this cell line. On the other hand, in U-251 cells this effect was less profound (Figure S2). Quantification of autophagy by flow cytometry displayed the most intensive process in LN-229 cultures exposed to both drugs confirming the hypothesis that autophagy induced by Lopi-NO is responsible for the reduced anticancer efficacy of Cisplatin (Figure 6B). This was further well synchronized with abrogated number of apoptotic cells detected by AnnV/PI double staining. 

## 3. Discussion

We have repeatedly demonstrated during the last years that covalent attachment of NO to the first HIV-PI Saq significantly boosted the anticancer action of the parental drug, retaining the antiviral potential and reducing the toxicity [14,15,32,39]. Subsequently, we generated in vitro data proving that the same chemical intervention applied to Lopi also resulted in intensified anticancer activity of Lopi-NO against hematological cancers [36]. However, no data has so far been generated on the efficacy of Lopi-NO to influence the growth of solid tumors.

To this aim, in the present study we first compared the effects of Lopi and Lopi-NO on the in vitro growth of LN-229 and U-251 human GBM cell lines. We have shown that the NO-hybridization significantly altered the antiglioma profile of Lopi. Indeed, Lopi-NO exerted twice stronger and different anticancer effects than Lopi. The curves of viability displayed specific shapes similar to those already observed in cell culture exposed to Saq-NO [14]. The occurrence of plateau-effect indicated that the action of Lopi-NO depended on the inhibition of cell proliferation rather than cell killing so that the toxicity of Lopi-NO did not correlate with further elevation of the dose. Concordantly, we found that treatment with Lopi-NO inhibited tumor cell proliferation that was not followed by significant impact of cell death. Moreover, while Lopi and Lopi-NO were equally potent in inhibiting cell division in LN-229 cells, only Lopi-NO possessed this potential in U-251 cells. We hypothesize that selective sensitivity of U-251 cells to the action of Lopi-NO may be secondary to the stronger chemotherapeutic action of Lopi-NO than Lopi that may have allowed overcoming some cellular mechanism of resistance of U-251 cells to Lopi. From a cellular and pharmacological point of view this is in accordance with the stronger intensity of autophagy induced by Lopi-NO in U-251 cells as well as with total caspase activity level. We hypothesize that the chemotherapeutic response to Lopi of the two GBM cell lines may be related to cell specificity and intrinsic difference between them. 

The GBM cell lines used in this study express similar panel of stem markers such as nestin, sex-determining region Y(SRY)–box 2 (Sox2), Musashi-1, and cluster of differentiation (CD)44 [38]. These cells exhibit higher migration and colonization abilities in comparison to other glioma cell lines. However, they differ in response to other environmental factors such as nutrition, indicating alteration in intracellular signature and ability to acquire specific phenotype [38]. While LN-229 can differentiate into neuronal cells, both neuronal and astrocytic differentiation was found in U-251 cell line. Cell maturation toward more specific phenotype is in general accompanied with the loss of dividing potential [14,15,40]. We found that withdrawal of Lopi-NO failed to restore cell viability, suggesting that it induced a permanent loss of proliferative properties. The proliferation inhibitory function is well-recognized mechanism of NO modified HIV-PIs that we previously observed with Saq-NO in melanoma, colon, prostate, and astrocytoma cells [14,33,40]. This property might be attributed to its potential of long-lasting interference with the activity of p70S6 kinase that is involved in the regulation of numerous proteins necessary for the cell division [14,32,33,36].

In the present study, the reduced proliferative capacity upon exposure to Lopi-NO was associated with changes of cell shape, which was more obvious in U-251 cells. Morphological transformation resembling astrocyte phenotype was previously determined in Saq-NO treated rat astrocytoma [14]. Estimation of GFAP expression revealed that Lopi-NO promoted strong astrocytic differentiation in U-251 but not in LN-229 cells. This result is in agreement with a previous study where differentiation potential of these two cell lines was estimated [38]. In the GFAP promotor, several binding sites for NO sensitive transcription factors exist, such as SP1, NFkB, etc. [41]. Mentioned factors are prone to s-nitrosylation mediated by NO [42,43]. Since our unpublished data indicate that Lopi-NO releases NO inside the cell, enhancement of GFAP expression can be ascribed to this NO-donating effects of Lopi-NO (Unpublished data). 

It has been reported that autophagy may be involved in the differentiation of glioma cells [44,45]. Since we detected amplified autophagy upon the treatment with Lopi-NO, it was interesting to explore the possible connection between this process and the astrocytic differentiation induced. However, cotreatment with 3-MA and Lopi-NO did not decrease GFAP expression indicating that autophagy was not pivotal in differentiation of U-251 cells. Furthermore, neutralization of autophagy did not modify the effect of Lopi-NO on cell viability in U-251 cells and even potentiated its action in LN-229 cells, suggesting that the autophagic process may represent a defensive response of GBM cell lines against the chemotherapeutic action of Lopi-NO. The role of autophagy in cancer has received great attention during the last years and conflicting evidence on whether it plays a pro- or anti-oncogenic role have been generated. The net effect may depend on tumor cell type and developmental phase of the tumor. However, chemotherapeutic effects of autophagy inhibitors have been described in vitro and in vivo. Our study suggests that combining Lopi-NO with autophagy inhibitors may prove beneficial for some forms of GBM. Similarly, inhibition of autophagy has been shown to increase susceptibility of GBM stem cells to Temozolomide by inducing ferroptosis [46,47,48].

Since production of ROS is important for cell proliferation, differentiation, and cell death, the influence of Lopi-NO on their generation was estimated in this study. Similarly to Saq-NO [33], Lopi-NO triggered the production of ROS/RNS. In agreement with the ability of Lopi-NO to release NO, it was expected that this highly reactive molecule might have contributed to the augmented anticancer action of Lopi-NO vs. Lopi. The analysis of cellular viability in the presence of intracellular NO scavenger, carboxy-PTIO, that recovered cell viability confirmed this hypothesis. 

One of the most important chemotherapeutic features of HIV-PIs is their sensitizing property. It was found that Lopi in combination with proteasome inhibitor Bortezomib/Carfizomib sensitized resistant myeloma cells [49]. Similarly, we published that Saq-NO can synergize with Cisplatin, Doxorubicin, or Paclitaxel in prostate cancer cell line PC3 [33]. The chemosensitizing properties of Saq-NO could be the consequence of its ability to inhibit the function of P-glycoprotein (P-gp), multidrug resistance gene 1 (MRP-1), and breast cancer resistance protein (BCRP-1) acting as a competitive inhibitor [50]. On the contrary, in this study combination of Lopi-NO with Cisplatin resulted in decreased Cisplatin toxicity toward LN-229 while this effect was less pronounced in U-251 cell line. This was manifested through decreased accumulation of early and late apoptotic cells in concomitant treatment in comparison to Cisplatin treated cultures. The reduction of Cisplatin cytotoxicity was in clear correlation with the intensity of autophagic process that was most intense in cultures exposed to both compounds. It has been previously demonstrated that autophagy inhibits apoptosis in Cisplatin-treated U-251 glioma, rat C6 glioma and mouse L929 fibrosarcoma cell lines [51]. Likewise, in A549 human lung cancer cells suppression of autophagy through inhibition of autophagy protein (Atg)5 and Beclin 1 forced apoptotic cell death triggered by Cisplatin [52]. Protective autophagy in response to Cisplatin may be responsible for the resistance development and treatment failure in bladder cancer cells, 5637 and T24 [53]. These data suggest that targeting of autophagy in parallel with the Lopi-NO treatment may increase the anticancer efficacy of Lopi-NO as we have presently observed in LN-229 cells.

Finally, an important pharmacological property of Lopi-NO was the capacity to induce differentiation of U-251 cells to an astrocyte lineage. These data are in agreement with the strong differentiation properties that we have previously observed with Saq-NO and indicate that induction of differentiation may be a common pharmacological property induced or potentiated by NO hybridization of HIV. This characteristic may play a role in the anticancer potential of NO-derived HIV-PI [15] as the differentiation toward nonmalignant cell phenotype has been studied for the treatment of cancer and could potentiate and prolong the effects of standard chemotherapeutic drugs [54].

It will be of interest to evaluate whether Lopi-NO is also able to inhibit the transdifferentiation properties of GBM cells toward mesenchymal or endothelial phenotype that has been associated with further acquirement of malignity and invasiveness of GBM and, for the latter, resistance to current standard of care therapy [55,56,57].

In conclusion, the presented data, extended to cancer cell lines representative of a solid tumor like GBM, revealed the augmented anticancer potential induced in Lopi by NO hybridization that we have previously shown in blood cancer cells [36]. The presently demonstrated superior chemotherapeutic potency of Lopi-NO vs. Lopi may have obvious important advantages for the long-lasting treatment of cancer patients in the clinical setting that may also relate to the use of reduced doses of Lopi-NO that may reduce incidence and severity of side effects observed with a full dose of Lopi that included metabolic dysfunction and cognitive impairment [58]. Along this line of research aimed at identifying synergistic combinations of Lopi-NO with other chemotherapeutic drugs we are currently carrying out in vitro studies of combined use of Lopi-NO with different anticancer drugs and also autophagy inhibitors that could further strengthen its chemotherapeutic profile. Finally, the effects of single and combined treatment of Lopi-NO with other in vitro identified synergistic anticancer compounds will be needed as well as in vivo models of GBM including xenograft and possibly orthotopic models to further confirm the potential chemotherapeutic action of Lopi-NO in GBM.

## 4. Materials and Methods

### 4.1. Reagents and Cells

Dulbecco’s modified Eagle’s medium (DMEM) was purchased from Biowest (Riverside, MO, USA). Fetal calf serum (FCS), phosphate-buffered saline (PBS), dimethyl sulfoxide (DMSO), carboxyfluorescein diacetate succinimidyl ester (CFSE), PI, CV, 3-MA, chloroquine, and carboxy-PTIO were obtained from Sigma (St. Louis, MO, USA). MTT was obtained from AppliChem (St. Louis, MO, USA). AnnV was from BD Pharmingen (San Diego, CA, USA) and apostat was from R&D Systems (Minneapolis, MN, USA). Paraformaldehyde (PFA) was purchased from Serva (Heidelberg, Germany). Acridine orange was from Labo-Moderna (Paris, France). Pen-Strep solution was from Biological Industries (Cromwell, CT, USA). Lopi was purchased from Hoffman-La Roche (Basel, Switzerland). Lopi-NO was obtained from OncoNox (Copenhagen, Denmark) and was synthesized as previously described [37].

The human GBM cell lines U-251 and LN-229 were purchased from the American Type Culture Collection (Rockville, MD, USA). Cells were propagated in HEPES-buffered high glucose DMEM medium, supplemented with 10% heat-inactivated FCS, 2 mM l-glutamine, 0.01% sodium pyruvate, penicillin (100 units/mL), and streptomycin (100 μg/mL) and incubated at 37 °C in a humidified atmosphere with 5% CO_2_. After standard trypsinization, cells were seeded overnight at 0.7 × 10^4^ cells/well in 96-well plates for viability determination and for cotreatment with Lopi-NO and Cisplatin, at 2.5 × 10^5^ cells/well in 6-well plates for flow cytometric analyses, at 0.3 × 10^4^ cells/well for Lopi-NO persistence determination and for immunocytochemistry in 4-chamber slide at 0.7 × 10^5^ cells/well.

### 4.2. Determination of Cell Viability by MTT and CV Assays

Lopi and Lopi-NO were dissolved in DMSO at a concentration of 10 mg/mL. Cells were treated for 48 h with seven scalar concentrations of Lopi-NO or its parental compound Lopi (1.56–100 µg/µL). Cell viability was evaluated using CV and MTT assays.

Moreover, to determine if the effect of Lopi-NO was persistent, U-251 and LN-229 cells were incubated for 120 h with the respective IC_25_ and IC_50_ doses of Lopi-NO. In parallel, the compound was removed from the culture after 48 h and cells were further cultivated for 72 h when the cell viability was measured by CV assay.

Furthermore, cells were pretreated for 24 h with IC_50_ of Lopi-NO and then Cisplatin was added. Viability was evaluated by CV assay after additional 24 h of cultivation in the presence of both drugs.

The MTT reduction assay evaluates cell respiration and cell viability through mitochondrial-dependent reduction of the tetrazolium salt MTT to the colored formazan product, which reflects the mitochondrial activity of cultured cells. After drug exposure, the cells were incubated with MTT solution (0.5 mg/mL) at 37 °C in a 5% CO_2_ atmosphere for approximately 1 h until formazan crystals were formed. Then, the supernatant was removed and 50 µL of DMSO was added to each well to dissolve produced formazan. The absorbance was measured on an automated microplate reader at 540/670 nm. The average values were determined from triplicate readings. The results of MTT assay were expressed as percentage compared to the control value obtained from untreated cell cultures, which was arbitrarily set to 100%.

CV assay was used to evaluate the number of adherent viable cells. After appropriate treatments, supernatant was discarded to remove nonadherent dead cells and the remaining cells were fixed with 4% PFA for 10 min at room temperature. Subsequently, the cells were stained with 2% CV solution for 15 min. Then, the plates were thoroughly washed with tap water and air-dried, and then at the end the dye was dissolved in 33% acetic acid. The absorbance of dissolved dye was measured at 540 nm with a reference wavelength of 670 nm on a microplate reader. Results were normalized to untreated cells and presented as % of control.

### 4.3. AnnV-/PI and Apostat Staining

The cells were treated with IC_50_ concentrations of either of Lopi or Lopi-NO for 48 h and then were stained with Ann V following the manufacturer’s instructions and PI (15 μg/mL) for 15 min at room temperature to detect the presence of apoptosis. In order to evaluate if apoptosis was caspase-dependent or not, the cells were stained with apostat according to manufacturer’s instructions. Cells were analyzed by CyFlow^®^ Space Partec using the PartecFloMax^®^ software (Partec GmbH, Münster, Germany).

### 4.4. CFSE Staining

For detection of cell proliferation, the cells were stained with CFSE (1 μM) for 10 min at 37 °C, then washed, seeded in 6-well plates, and exposed to IC_50_ dose of Lopi and Lopi-NO for 48 h. At the end the cells were detached, washed, resuspended in PBS, and analyzed with CyFlow^®^ Space Partec using the PartecFloMax^®^ software.

### 4.5. Acridine Orange Staining

To detect the presence of acidic vesicles as a mark of autophagy, the cells were stained with a solution of 10 μM acridine orange during 15 min at 37 °C, after exposure to either Lopi or Lopi-NO during 48 h, then they were washed and resuspended in PBS and finally analyzed with CyFlow^®^ Space Partec using the PartecFloMax^®^ software.

### 4.6. Measurement of ROS and RNS

To detect reactive oxygen and nitrogen species, U-251 cells were prestained with 1 μM DHR (Molecular Probes, Eugene, OR, USA) for 20 min at 37 °C, seeded in 6-well plates, and then treated with IC_50_ dose of Lopi-NO. After 48 h period of incubation, cells were trypsinized and analyzed with CyFlow^®^ Space Partec using the PartecFloMax^®^ software. 

### 4.7. Immunocytochemistry

The cells were seeded in 4-chamber slide and incubated with the IC_50_ dose of Lopi-NO for a 48 h period in order to detect possible signs of differentiation process. At the end of incubation, the cells were fixed with 4% PFA for 20 min at room temperature and the cell membrane was permeabilized with 0.5% Triton X-100 in PBS (PBST) for 30 min. Subsequently, the activity of endogenous peroxidases was inactivated by 3% H_2_O_2_ with10% methanol in 0.5% PBST for 10 min and unspecific binding sites were blocked using 5% FCS in 0.1% PBST for 1 h at room temperature in wet chamber. After washing with PBS, the cells were incubated with primary antibody GFAP (Dako North America, Carpinteria, CA, USA) diluted in 1% bovine serum albumin (BSA), BSA-PBS at 4 °C overnight in wet chamber. As negative control, 1% BSA-PBS without primary antibody was used. The detection of reaction antigen-primary antibody was done using ExtrAvidin Peroxidase Staining kit (Sigma, St. Louise, MO, USA) according to manufacturers’ instructions. As a substrate for peroxidase, 3,3′-diaminobenzedine (DAB, Dako North America, Carpinteria, CA, USA) was used. After the color had developed, the staining reaction was stopped with PBS. Contrast staining was performed using filtered Mayer’s hematoxylin for approximately 30–60 s. The excess stain was removed by shortly rinsing with tap water. The slide was prepared for further light microscopy analysis by covering with glycergel mounting medium (Dako North America, Carpinteria, CA, USA).

### 4.8. Statistical Analysis

The results of cellular viability are expressed as mean ± standard deviation (SD) from three independent experiments. We used the Statistica 12 Package for data analysis. A Student *t*-test was employed to evaluate the significance between groups. A *p* value less than 0.05 was considered to be statistically significant.

## Figures and Tables

**Table 1:** IC_50_ values of Lopi and Lopi-NO in GBM cell lines. Data are presented as mean ± standard error of the mean (SEM) of three independent experiments.

Cell Line	Assay	IC50 (µg/mL)	
		Lopinavir	Lopinavir-NO
LN-229	MTT	13.80 ± 2.40	8.70 ± 1.41
	CV	13.75 ± 2.90	7.30 ± 1.70
U-251	MTT	21.50 ± 0.85	13.30 ± 1.13
	CV	22.35 ± 0.07	12.50 ± 0.00

## Supplementary Materials

Figure S1: Cytoprotective role of autophagy in Lopi-NO treated LN-229 cells. Cells were treated with IC_50_ value of Lopi-NO in the presence of autophagy inhibitor 3-MA (1 mM) or chloroquine (20 μM) for 48 h and cellular viability by MTT test was estimated. * *p* < 0.05 compared to untreated cultures, Figure S2: Interplay between Lopi-NO and Cisplatin on U-251 cells. Cells were exposed to Cisplatin in the presence of IC_50_ value of Lopi-NO and cellular viability was measured by CV test.
