# Supplementary material for: Anticancer and Differentiation Properties of the Nitric Oxide Derivative of Lopinavir in Human Glioblastoma Cells
Source: Molecules. 2018 Sep 26;23(10):2463. doi: 10.3390/molecules23102463 (PMC6222694; doi:10.3390/molecules23102463)
Supplement: Supplementary file 1 [file molecules-23-02463-s001.pdf]

Article

Anticancer and differentiation properties of the nitric-oxide derivative of the HIV protease inhibitor Lopinavir in human glioblastoma cells

Maria Sofia Basile [1,2], Emanuela Mazzon [3], Tamara Krajnovic [1], Dijana Draca [1], Eugenio Cavalli [3], Yousef Al-Abed [4], Placido Bramanti [3], Ferdinando Nicoletti [2,*], Sanja Mijatovic [1] and Danijela Maksimovic-Ivanic [1]

[1] Department of Immunology, Institute for Biological Research "Sinisa Stankovic", Belgrade University, Bulevar despota Stefana 142, 11060 Belgrade; Serbia; sofiabasile@hotmail.it; tamara_krajnovic@yahoo.com; dracadiana@gmail.com; sanjamama@yahoo.com; nelamax@yahoo.com

[2] Department of Biomedical and Biotechnological Sciences, University of Catania, Via S. Sofia 89, 95123 Catania, Italy; sofiabasile@hotmail.it

[3] IRCCS Centro Neurolesi Bonino Pulejo, Strada Statale 113, C.da Casazza, 98124 Messina, Italy; emanuela.mazzon@irccsme.it; eugeniocavalli9@hotmail.it; placido.bramanti@irccsme.it

[4] Center for Molecular Innovation, The Feinstein Institute for Medical Research, 350 Community drive, Manhasset, NY, 11030, USA; yalabed@northwell.edu

* Correspondence: E-mail address: ferdinic@unict.it; Tel.: +390954781270

Received: 31 August 2018; Accepted: date; Published: date

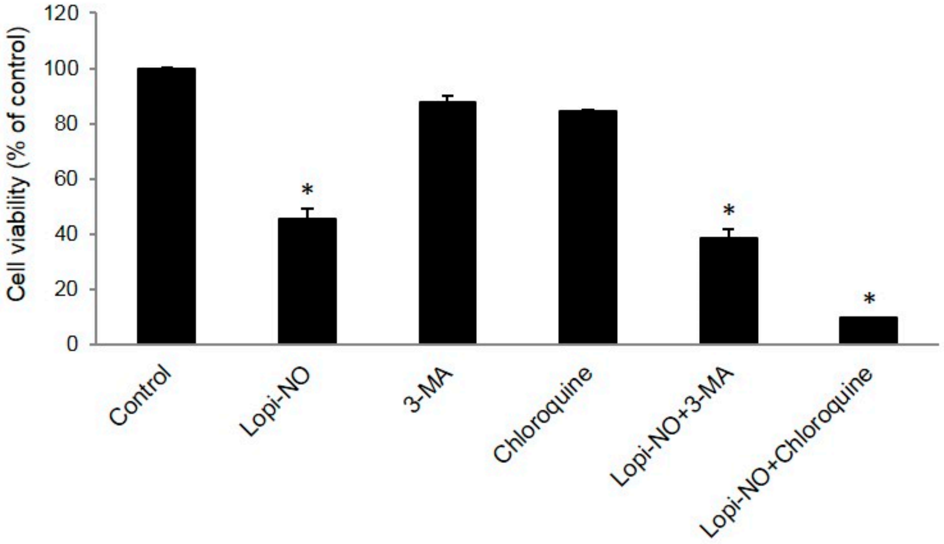

Figure S1. Cytoprotective role of autophagy in Lopi-NO treated LN-229 cells. Cells were treated with IC_{50} value of Lopi-NO in the presence of autophagy inhibitor 3-MA (1 mM) or chloroquine (20 μM) for 48 h and celluar viability by MTT test was estimated. * $p < 0.05$ compared to untreated cultures.

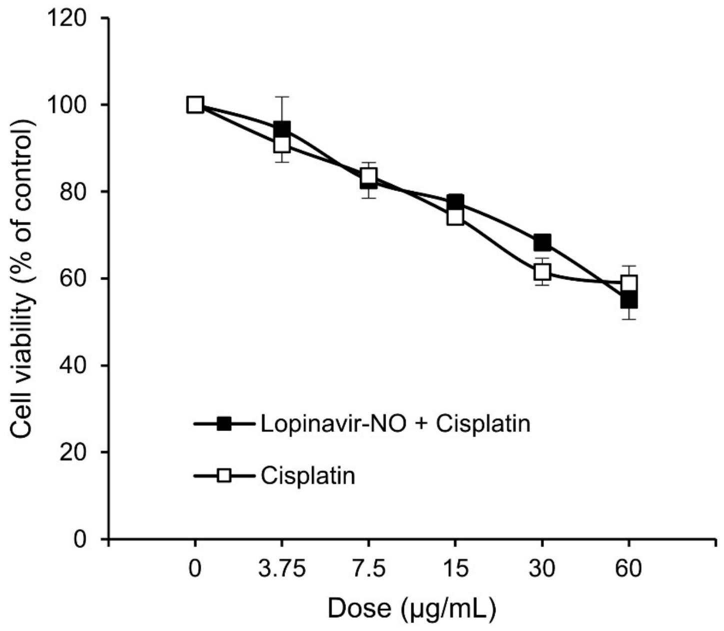

Figure S2. Interplay between Lopi-NO and cisplatin on U-251 cells. Cells were exposed to cisplatin in the presence of IC_{50} value of Lopi-NO and cellular viability was measured by CV test.
